# Individuals with Down Syndrome: Editorial

**DOI:** 10.3390/brainsci12030398

**Published:** 2022-03-16

**Authors:** Silvia Lanfranchi, Chiara Meneghetti, Enrico Toffalini, Barbara Carretti

**Affiliations:** 1 Department of Developmental and Social Psychology, University of Padova, 35131 Padova, Italy; silvia.lanfranchi@unipd.it; 2 Department of General Psychology, University of Padova, 35131 Padova, Italy; enrico.toffalini@unipd.it

Down syndrome (DS) is the most common syndromic cause of intellectual disability, so it has long been of interest to researchers. Although, on average, individuals with DS reach a cognitive functioning level equivalent to that of five- to eight-year-old children, they have distinctive patterns of relative strengths and weaknesses. For a long time, individuals with DS have been described as relatively strong in terms of visuospatial abilities and relatively weak in the sphere of verbal skills [1]. The literature—including the studies collected in this Special Issue—has shown, however, that this may be an overgeneralization. Several studies have also identified peaks and troughs in the DS profile within each of these domains. As concerns verbal skills, for instance, individuals with DS were better in vocabulary, intentional use of communication and gestures, and social use of communication, than in phonology, grammar, and syntax [2,3]. More in general, their receptive language tends to be less impaired than their productive language [4]. A pattern of strengths and weaknesses has emerged in the visuospatial domain as well, as individuals with DS are less impaired in terms of spatial–sequential working memory and visuo-motor integration than they are in visual and spatial–simultaneous working memory, and in mental rotation, although the degree of rotation can make a difference [5,6]. Attention has also turned more recently to inter-individual variability in the DS profile. For example, when Karmiloff-Smith and colleagues [7] conducted a review, they noted in a number of studies exploring different domains that the variability within the group of individuals with DS ranged from two to three times the variability seen in groups of typically developing individuals. 

Continuing in this vein, this Special Issue contains eight empirical articles that aim to shed more light on the cognitive profiles of individuals with DS by focusing mainly on the predictors of the cognitive abilities and their developmental trajectories. Most of the studies are devoted to understanding which factors might play a part in explaining individual differences in the cognitive functioning of individuals with DS. 

Focusing on infancy, Schworer and colleagues [8] longitudinally examined a group of infants with DS in terms of the relationship between their early regulatory functions and their communication abilities, assessing the former at T1, and using the findings to predict the latter at T2. Early regulatory functions (specifically, the Orienting/Regulation dimension of the Infant Behavior Questionnaire-Revised) at 9 months of age were found to predict later social communication abilities (assessed with the Communication and Symbolic Behavior Scales—Infant Toddler Checklist) at 15 months, even after controlling for general cognitive ability and prematurity. Generally speaking, children with DS have good socializing skills, but this study identified a substantial variability in their communication and language outcomes and demonstrated that measuring regulatory functions enables social communication difficulties to be detected—and thus addressed—in infancy. 

The early stages of development in individuals with DS, and especially the age at which they reach certain milestones, have also been demonstrated to correlate with their subsequent development in preschool and school years. Locatelli and colleagues [9] examined the association between the age at which children reached early milestones such as sitting, babbling, walking, or sphincter control and their later development in preschool and school age. They found that sitting predicted motor development, and babbling predicted language development, while sphincter control emerged as the strongest predictor of numerous abilities, including motor, general cognitive, language, and adaptive skills, in both preschool and school age. This would suggest that the same neural substrate is shared by these milestones and later general cognitive development. More in general, investigating early milestones may have important applied implications for the clinical assessment of children with DS, to gather information on their likely future cognitive growth.

Focusing on primary school age, Næss and colleagues [10] examined vocabulary development trajectories longitudinally in six-year-old children with DS, assessed at three time points: at 76 months old (T1), then 12 months later (T2), and after another 12 months (T3). A control group of typically developing (TD) children of similar age was also tested. Numerous variables encompassing nonverbal mental ability, receptive vocabulary, auditory memory, phonological awareness, oral motor skills, home literacy environment, socio-economic status, and socioemotional functioning were considered as predictors. The growth curve was less steep for the children with DS than for the TD children. Several predictors of expressive vocabulary development were identified for both groups, including home literacy environment, receptive vocabulary, and auditory memory, even after controlling for sex, parental education, and nonverbal mental age. It was only for individuals with DS, however, that expressive vocabulary development was also predicted by phonological awareness and oral motor skills. Individuals with DS are frequently weak in the latter, and this might impair their verbal articulation and acquisition of new words. In short, oral motor skills may represent an important variable in this population’s vocabulary development.

Developmental trajectories have also been described for the visuospatial domain, focusing mainly on spatial visualization and mental rotation abilities. In their sizable cross-sectional study, Doerr and colleagues [11] examined a group of individuals with DS from 8 to 53 years old, considering the changes in these domains. They tested both linear and non-linear (segmented) models constructed to describe this development. It emerged that spatial visualization ability did not vary substantially with chronological age beyond 8 years old, although it remained related to equivalent age in the Raven’s Colored Progressive Matrices (CPM). Mental rotation skills continued to improve up to 14 years of chronological age, then stabilized, and were also positively related to participants’ CPM scores, especially for those achieving higher levels of performance. Analyzing items of the mental rotation task separately suggested that the difficulty of a given item was important, as children with DS are unable to manage 180° of mental rotation. In general, however, it seems that individual differences tend to prevail over chronological age difference in DS, even at the developmental stage.

Similarly, the study by Fontana and colleagues [12] suggests that older adults with DS perform better than younger ones in executive control tasks. The authors focused on response inhibition, interference suppression, and delayed gratification. They found participants with DS more impaired overall by comparison with TD children with the same nonverbal reasoning ability in executive control. The former had deficits in all three areas considered, suggesting that executive control might be a particular problem in DS. 

Another aspect that might explain individual differences in the DS profile concerns the role of the environment. With this in mind, Ranzato and colleagues [13] examined cognitive profiles in relation to a participant’s home environment, including home learning activities (e.g., playing dominoes for mathematical skills, playing games that promote reading, talking about money when shopping), the use of technology (such as a computer), and parents’ attitudes. Primary-school children with DS were assessed and compared with children with Williams syndrome (WS) of similar age and general cognitive functioning (apart from expressive abilities). On average, the children with DS had weaker expressive verbal abilities and thus needed more visual support than their WS peers during home learning activities. The two groups’ home environments were generally similar, and such environments reflected the individuals’ cognitive profiles more than their specific syndrome. There were also no differences between the DS and WS groups in terms of the parents’ expectations regarding their children’s academic abilities. Overall, more time was spent on literacy-based activities than on numeracy-based activities, but there were relatively more counting and number recognition activities for children with DS than for those with WS, despite the two groups presenting similar difficulties. The environment, and particularly the literacy of the home environment, has a role in vocabulary development in school-age children, as also seen in the above-mentioned study by Næss and colleagues [10].

More in general, the environment—seen as the context in which a particular skill is required—might play a part in modulating how children express that particular skill. To this end, Yang et al. [14] examined episodic memory functioning in DS, focusing on both daily life abilities and lab-based performance. They found that, when tested with lab-based tasks, individuals with DS were more impaired than individuals (of similar or superior mental age) with intellectual disabilities (ID) of mixed etiology when it came to long-term memory, but the same did not apply to everyday memory. Participants were tested on the latter with a series of items such as recalling their own phone number, street name, or bedtime, and their answers were compared with those of a parent to assess their accuracy. Despite individuals with DS presenting this mismatch between the two types of long-term (episodic) memory, when compared with the mixed-etiology ID group, performance in standardized lab-based tasks was still positively associated with everyday memory performance in each of the two groups, and it was so even after controlling for general cognitive ability. This study also offers insight into the importance of considering the type of material to be memorized: in this case, remembering things relating to their everyday lives is probably easier for individuals with DS than recalling lab-based material.

Finally, Roch and colleagues [15] examined reading comprehension in relation to reading speed and accuracy, receptive vocabulary, and listening comprehension, adopting the theoretical framework of the “simple view of reading”, according to which reading comprehension involves two basic components: word recognition/decoding, and language comprehension. Young adults with DS were compared with TD children and individuals with autism spectrum disorder (ASD) with comparable levels of reading accuracy and receptive vocabulary. In both the atypical groups (DS, ASD), reading comprehension was only predicted consistently by listening comprehension, and to a lesser degree by vocabulary or basic reading skills (reading speed and accuracy). In short, the variables considered by the simple view of reading adequately predicted the reading comprehension profile of the individuals with DS, but the reading comprehension predictors were not equally important in such an atypical population (i.e., listening comprehension was a stronger predictor than basic reading skills in the case of DS). In fact, both the atypical groups behaved as poor comprehenders with typical development, i.e., their reading skills were better than their reading comprehension, the latter being constrained by language comprehension. This study paves the way to future investigations on the reading strategies and comprehension processes of individuals with DS.

Taken together, the contributions in this Special Issue further emphasize the importance of focusing not only on average patterns of strengths and weaknesses in the DS profile but also on inter-individual variability within this population. This demands longitudinal or cross-sectional studies investigating a broad range of basic and complex cognitive abilities, and everyday life skills.

Taking the developmental trajectories approach enables us to detect the precursors of a given skill, and this will help clinicians to target them effectively in early interventions to establish strong foundations for the later development of more complex abilities. This approach can also reveal variables that might play a part in facilitating or hampering the development of a given skill, enabling us to identify children at risk of developing particular problems. We believe that all these aspects may be of particular interest to clinicians striving to develop effective customized intervention programs that capitalize on an individual’s strengths in order to help them overcome the obstacles to their achievement of a better quality of life.

On the methodological side, future challenges will concern how to refine the sampling and recruitment of participants (studies generally rely on convenience samples of uneven size), to improve statistical and methodological practices (none of the studies were preregistered, and the criteria for assessing the level of evidence were heterogeneous), to expand the use of longitudinal data collection, and to improve the modelling (even studies with longitudinal data failed to perform latent growth modelling to study individual trajectories, for example). More attention will also have to be devoted to specific processes behind cognitive abilities. That said, the present Special Issue provides an up-to-date account of the most recent lines of research on individuals with DS and offers researchers a set of findings that can serve as starting points for future studies.
